# Linking anti-Ro antibodies to fibrosis in congenital heart block: translation to a clinical trial

**DOI:** 10.1186/ar4631

**Published:** 2014-09-18

**Authors:** Jill P Buyon, Peter Izmirly, Deborah Friedman, Joshua Copel

**Affiliations:** 1 New York University School of Medicine, New York, USA; 2 New York Medical College, Valhalla, NY, USA; 3 Yale University School of Medicine, New Haven, Ct, USA

## Background

One of the strongest clinical associations with autoantibodies (Ab) directed to components of the SSA/Ro-SSB/La ribonucleoprotein complex is the development of congenital heart block (CHB) in an offspring. Fetal disease is independent of maternal disease and often anti-Ro Ab are first sought only because CHB has been identified. The first-time risk of 2% is 10-fold higher in women who have had a previous CHB child. Tissue injury in the fetus is presumed to be dependent on the FcγR-mediated transplacental passage of maternal IgG Ab. Despite attempts of large multicenter studies to forestall disease by careful monitoring, irreversible block and extensive myocardial injury have been documented within 7 days of a normal rhythm and PR interval. *In vivo *and *in vitro *data support that cardiac fibrosis may be consequent to macrophage Toll-like receptor (TLR) signaling following ligation of the ssRNA complexed to the Ro protein. TLR signaling and fibrotic endpoints may be abrogated by chloroquine (CQ), which inhibits endosomal acidification. This *in vitro *observation was initially "translated" to patients by evaluating the use of hydroxychloroquine (HCQ) in an extensive case-control retrospective chart review of anti-Ro/La Ab exposed fetuses of mothers with SLE enrolled in three databases. This approach was followed by another study that addressed whether HCQ use reduces the expected recurrence rate of CHB. The collective results were highly encouraging.

## Methods and results

Based on these studies we initiated an open-label prospective study design using Simon's two-step approach. HCQ at 400 mg is initiated by 10 weeks following conception. Serial echocardiograms (monitor PR interval) and evaluation of maternal and cord blood biomarkers (HCQ levels, IFNα signatures, and Ab titers) are part of the protocol to address maternal compliance, pathobiology and efficacy. The first stage has been completed with 19 subjects and only one recurrence of CHB.

## Future directions

Over the next 4 years, 35 subjects will be enrolled. Ultimately, HCQ will be considered efficacious for the prevention of CHB if fewer than six cases occur among a total of 54 subjects evaluated. A positive result will probably change the management of all anti-Ro-positive women who have had a previous child with CHB and illustrate the importance of translational science. Perhaps most relevant to impact, a potential prevention would justify screening of all pregnant women for anti-Ro antibodies, particularly relevant since mothers of affected children are frequently asymptomatic, a point critical to early pregnancy counseling.

